# Phenolics and Terpenoids Profiling in Diverse Loquat Fruit Varieties and Systematic Assessment of Their Mitigation of Alcohol-Induced Oxidative Stress

**DOI:** 10.3390/antiox12101795

**Published:** 2023-09-23

**Authors:** Qun-Jiao Yan, Yun-Yi Chen, Man-Xi Wu, Han Yang, Jin-Ping Cao, Chong-De Sun, Yue Wang

**Affiliations:** 1 Institute of Health Service and Transfusion Medicine, Academy of Military Medical Sciences, Academy of Military Sciences, Beijing 100000, China; yanqunjiao19@mails.ucas.ac.cn; 2 Laboratory of Fruit Quality Biology, The State Agriculture Ministry Laboratory of Horticultural Plant Growth, Development and Quality Improvement, Zhejiang University, Zijingang Campus, Hangzhou 310000, China; 12016039@zju.edu.cn (Y.-Y.C.); mxwu@zju.edu.cn (M.-X.W.); 22116154@zju.edu.cn (H.Y.); 0017165@zju.edu.cn (J.-P.C.); adesun2006@zju.edu.cn (C.-D.S.)

**Keywords:** loquat, phenolics, terpenoids antioxidant, alcohol-induced oxidative stress

## Abstract

To compare and investigate the phenolic compounds in the peel and flesh of loquat (*Eriobotrya japonica*) and evaluate their ability to protect against alcohol-induced liver oxidative stress, we employed a combination of ultra-performance liquid chromatography (UPLC) and high-resolution mass spectrometry (HRMS) to qualitatively and quantitatively analyze 22 phenolics and 2 terpenoid compounds in loquat peel and flesh extracts (extraction with 95% ethanol). Among these, six compounds were identified for the first time in loquat, revealing distinct distribution patterns based on variety and tissue. Various chemical models, such as DPPH, FRAP, ORAC, and ABTS, were used to assess free radical scavenging and metal ion reduction capabilities. The results indicate that peel extracts exhibited higher antioxidant capacity compared with flesh extracts. Using a normal mouse liver cell line, AML-12, we explored the protective effects of loquat extracts and individual compounds against ethanol-induced oxidative stress. The findings demonstrate the enhanced cell viability and the induction of antioxidant enzyme activity through the modulation of *Nrf2* and *Keap1* gene expression. In a C57/BL6 mouse model of alcohol-induced liver damage, loquat extract was found to alleviate liver injury induced by alcohol. The restoration of perturbed serum liver health indicators underscored the efficacy of loquat extract in reclaiming equilibrium. The culmination of these findings significantly bolsters the foundational knowledge necessary to explore the utilization of loquat fruit extract in the creation of health-focused products.

## 1. Introduction

Loquat (*Eriobotrya japonica*), a perennial fruit tree belonging to the *Eriobotrya* genus in the Rosaceae family, possesses both medicinal and edible properties. The natural products derived from loquat have found applications in the treatment and prevention of various chronic diseases and suboptimal health conditions [1,2,3]. While initial investigations into loquat primarily centered on its leaves [2] and flowers [1], the biological activities of its fruits and the key active compounds responsible for these effects require further exploration. Specifically, further investigations are needed to understand the differences in bioactive substances and their activities between the distinct red-fleshed and white-fleshed loquat fruits. Previous studies have conducted preliminary testing on the chemical composition of the loquat fruit and its antioxidant activity. For example, Ferreres et al. identified 18 compounds in leaves and fruits (peel and flesh) of 6 improved cultivars of loquat, including 8 hydroxycinnamic acid derivatives and 10 flavonoid glycosides by HPLC-DAD-ESI-MS/MS, and conducted quantitative analysis on these compounds [4]. Their research focused on phenolic compounds and the testing of DDPH scavenging capacity using a variety with the highest content of phenolic compounds. Similar studies have also been recently conducted. Zhang et al. depicted the phytochemical profile of different parts of loquat using UHPLC-QTOF-MS, and tentatively annotated 349 compounds, including flavonoids, phenolic acids, lignans, stilbenes, and terpenoids [5]. However, their study did not involve quantitative analysis and did not differentiate between the fruit peel and the fruit flesh. In addition, they used only chemical models, such DPPH, ABTS, FRAP et al., to evaluate the in vitro antioxidant activities of different loquat extracts.

In recent years, the rise in global alcohol consumption has resulted in high-risk drinking becoming the fifth leading risk factor for world healthcare, leading to pathophysiological consequences in multiple organs and systems of the human body [6]. The liver plays a crucial role in alcohol metabolism, as about 90% of alcohol undergoes oxidation within the liver, making it the primary organ affected by alcohol-related damage [7]. Alcoholic liver injury (ALI) is a dynamic process characterized by impaired liver cell function caused by alcohol. As alcohol intake increases, this process shifts from a reversible physiological state to an irreversible pathological state. ALI can progress gradually and cause conditions such as alcoholic fatty liver (AFL), alcoholic hepatitis (AH), alcoholic hepatic fibrosis (AHF), alcoholic cirrhosis (AC), and even hepatocellular carcinoma (HCC) [8]. According to the Global Burden of Disease study, approximately 1,256,900 deaths can be attributed to cirrhosis and chronic liver injury, with around 334,900 (27%) of those linked to alcohol consumption [9]. Additionally, roughly 245,000 deaths were caused by alcohol-induced liver cancer, accounting for 30% of all liver cancer-related deaths [10].

Currently, there are drugs available for the treatment of ALI; however, their reported effectiveness varies, and some medications may cause gastrointestinal and renal toxic side effects [11]. Because there is no specific targeted therapy for ALI, the main focus of prevention and treatment revolves around alcohol abstinence and nutritional support. Natural products present several advantages, such as low toxicity, multi-pathway action, and broad targets, making them valuable resources for the development of health products. In light of the challenges in global public health, conducting thorough research and exploring the potential of natural products for the prevention and treatment of ALI holds great scientific importance.

Emerging from previous research, loquat has demonstrated various biological effects, encompassing hyperlipidemia, hyperglycemia, hypoglycemic, anticancer, and inhibition of non-alcoholic liver disease [3]. However, the preventive and therapeutic effects of loquat on ALI, particularly its role in ameliorating alcohol-induced liver oxidative stress, remain incompletely understood. Therefore, our study postulated that active compounds in loquat might exhibit inhibitory effects on alcoholic liver injury through antioxidant mechanisms. To substantiate this hypothesis, we performed a comprehensive analysis of the active substances in loquat and compared the contents of loquat fruit extracts from different varieties and parts. Using chemical methods and both in vitro and in vivo models, we conducted a thorough evaluation of the antioxidant capacity and ALI inhibitory potential of loquat fruit extracts. The research findings illuminate the way in which loquat may serve as an exceptional source of antioxidants for the prevention and treatment of ALI. Loquat effectively scavenges free radicals, reduces metal ions, and activates antioxidant pathways to modulate the activity of antioxidant enzymes, thereby mitigating the stress imposed on liver cells by alcohol. This study represents the first exploration of the preventive and therapeutic effects of loquat fruit extracts on alcohol-induced liver oxidative stress, providing a scientific foundation by which to harness the potential of loquat and develop unique health resources from food sources.

## 2. Materials and Methods

### 2.1. Materials and Chemicals

**Figure 1:**
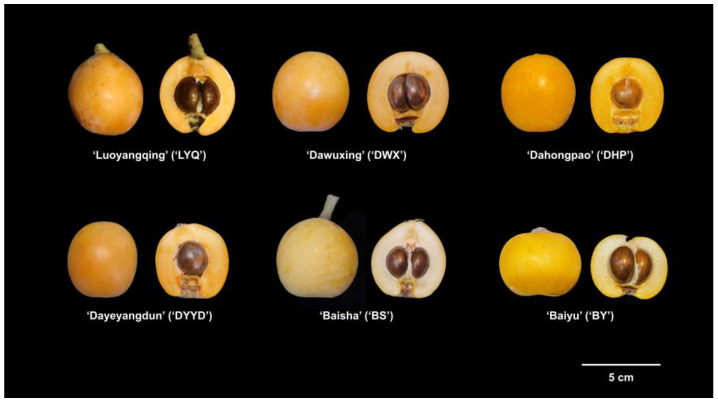
Six varieties of loquat fruits used in the study.

Six varieties of loquat fruits were selected for the experiment, namely ‘Luoyang Qing’ (LYQ), ‘Da Wu Xing’ (DWX), ‘Da Hong Pao’ (DHP), ‘Da Ye Yang Dun’ (DYYD), ‘Bai Yu’ (BY), and ‘Bai Sha’ (BS). Four of these were red-fleshed (‘LYQ’, ‘DWX’, ‘DHP’, ‘DYYD’), while two were white-fleshed (‘BY’, ‘BS’) (Figure 1). All loquat samples were sourced from a loquat germplasm nursery located in Hangzhou, Zhejiang, China. The maturity period of the six varieties of loquat in the germplasm nursery is approximately May 10th to 30th, and the harvest time of the loquats used in this study is 21 May 2022. After careful selection, loquat fruits with an intact shape and no mechanical or insect damage were promptly transported to the laboratory within 6 h.

Standards, including 3-caffeoylquinic acid, 3-*p*-coumaroylquinic acid, 5-caffeoylquinic acid, 4-*O*-caffeoylquinic acid, sinapic acid, procyanidin B2, ferulic acid-4-*O*-glucoside, 1-*O*-caffeoylquinic acid, caffeic acid, procyanidin C1, 5-feruloylquinic acid, quercetin-3-*O*-neohesperidoside, quercetin-3-*O*-sambubioside, quercetin-3-*O*-galactoside, kaempferol-3-*O*-neohesperidoside, kaempferol-3-*O*-glucoside, quercetin-3-*O*-rhamnoside, kaempferol-3-*O*-rhamnoside, oleanolic acid, and ursolic acid were purchased from Shanghai yuanye Bio-Technology Co., Ltd. (Shanghai, China) DMEM/F12 cell culture medium, trypsin, penicillin, streptomycin, amphotericin B, and fetal bovine serum (FBS) were purchased from Gibco (Waltham, MA, USA). Cell Counting Kit-8 (CCK-8) was purchased from Dojindo Molecular Technologies, Inc. (Shanghai, China). DCFH-DA probe (S0033), CAT (S0051), SOD (S0109), GPx (S0056) kits for the detection of cellular ROS and antioxidant enzyme activity were purchased from Beyotime Institute of Biotechnology (Hangzhou, China). ALT (C009-3-1), AST (C010-3-1), HDL-C (A112-1-1), LDL-C (A113-1-1), TC (A111-1-1), TG (A110-1-1), and MDA (A003-4-1) kits for the detection of animal serum and liver indexes were purchased from Nanjing Jiancheng Bioengineering Institute (Nanjing, China). Trizol, PrimeScript™ RT reagent Kit with gDNA Eraser, and SsoFast™ EvaGreen^®^ Supermix kit were purchased from Takara Bio Inc (Dalian, Chian). The SPE C18 columns were purchased from Waters (Milford, MA, USA). Acetonitrile was chromatographically pure and purchased from Sigma Aldrich (St. Louis, MO, USA). The remaining reagents were analytically pure, purchased from Sinopharm (Beijing, China).

### 2.2. Extraction Method for Loquat Flesh and Peel Extracts

After cleaning the fruits with purified water, they were carefully separated into peel and flesh parts and immediately frozen in liquid nitrogen. The samples were then subjected to freeze-drying in a vacuum freeze-dryer and stored at −70 °C in a refrigerator for subsequent experiments. The freeze-dried powder of loquat flesh and peel (1 g) was individually mixed with 20 mL of 95% ethanol at 25 °C for ultrasonic-assisted extraction. The resulting supernatant was collected by centrifugation at 10,000 rpm for 5 min. The supernatant was then subjected to a second round of precipitation and extraction with 20 mL of 95% ethanol, and this extraction process was repeated three times. The crude extracts of loquat fruit samples were evaporated to complete dryness using a rotary evaporator and subsequently dissolved in deionized water, yielding the loquat fruit sample solution.

Next, the loquat fruit sample solution underwent purification using a Waters SPE C18 column, involving five sequential steps: activation, equilibration, sample loading, washing, and elution. To activate the SPE column, 12 mL of methanol was used, and was then equilibrated with 12 mL of deionized water. Following this, the loquat fruit sample solution (20 mL) was carefully introduced into the SPE column at a flow rate of less than 1 mL/min. After sufficient adsorption, the column was washed with 20 mL of deionized water to remove polar substances such as sugars and acids present in the fruit. Once the washing step was completed, the adsorbed phenolics and terpenoids were eluted from the column using analytical-grade methanol. The eluate was then subjected to rotary evaporation at 50 °C until its volume reduced to less than 1 mL, and the concentrated solution was transferred to a 2 mL centrifuge tube. Finally, the tube was placed in a vacuum centrifuge at 40 °C to evaporate the solvent, resulting in the enriched extraction of phenolics and terpenoids.

### 2.3. Identification and Quantification of Phenolics and Terpenoids in Loquat Fruits

The analysis of phenolic and terpenoids was performed using an ultra-high-performance liquid chromatography (UPLC) system coupled with a Triple-TOF 5600 high-resolution mass spectrometer (UPLC-ESI-MS/MS) (Waters, Milford, MA, USA). In the UPLC system, a Waters BEH C18 (Acquity UPLC, 2.1 × 150 mm) column was used as the stationary phase, with water (mobile phase A) and acetonitrile (mobile phase B) as the mobile phases. The gradient elution program was as follows: 0–2 min, 5% B; 2–8 min, 5–28% B; 8–12 min, 28–60% B; 12–14 min, 60–100% B; 14–14.5 min, 100% B; 14.5–15 min, 100–5% B. The detection wavelength was set at 280 nm, the flow rate was 0.15 mL/min, the column temperature was maintained at 25 °C, and the injection volume was 2 μL. Mass spectrometry conditions in the UPLC-Triple-TOF 5600+ high-resolution mass spectrometer were as follows: positive and negative ion scanning modes; scan range: *m*/*z* 100–1500; gas 1 (GS1) and gas 2 (GS2) set at 55 psi; curtain gas (CUR) at 35 psi; ion source temperature (TEM) set at 600 °C (positive mode) and 550 °C (negative mode); ion source voltage (IS) at 5500 V (positive mode) and −4500 V (negative mode). For the first-level scan, the declustering potential (DP) was set at 100 V, and the collision energy (CE) was set at 10 V. For the second-level scan, TOF MS–Product Ion–IDA mode was used to collect mass spectrometry data, with a collision-induced dissociation (CID) energy of 40 ± 20 eV. Prior to sample injection, a continuous direct infusion of calibration solution (CDS) was performed to achieve mass axis calibration and ensure a mass error of less than 2 ppm.

Among the identified compounds, 3-caffeoylquinic acid, 3-*p*-coumaroylquinic acid, 5-caffeoylquinic acid, 4-*O*-caffeoylquinic acid, sinapic acid, procyanidin B2, ferulic acid-4-*O*-glucoside, 1-*O*-caffeoylquinic acid, caffeic acid, procyanidin C1, 5-feruloylquinic acid, quercetin-3-*O*-neohesperidoside, quercetin-3-*O*-sambubioside, quercetin-3-*O*-galactoside, kaempferol-3-*O*-neohesperidoside, kaempferol-3-*O*-glucoside, quercetin-3-*O*-rhamnoside, kaempferol-3-*O*-rhamnoside, oleanolic acid, and ursolic acid were compared with standard products, and the contents were determined using the chromatographic peak area standard curve method. The contents of the remaining compounds were determined relative to the peak area of 3-caffeoylquinic acid.

### 2.4. Chemical Antioxidant Evaluation Methods

The loquat peel (YQ-peel, DWX-peel, DHP-peel, DYYD-peel, BY-peel, BS-peel) and flesh (LYQ-flesh, DWX-flesh, DHP-flesh, DYYD-flesh, BY-flesh, BS-233 flesh) extracts obtained in Section 2.2 were used for antioxidant evaluation in this part.

DPPH radical scavenging capacity: Two microliters (2 μL) of appropriately diluted sample were added to 198 μL of a freshly prepared 60 μM DPPH solution. The mixture was allowed to react in the dark at 25 °C for 2 h. The absorbance at 517 nm was measured using a microplate reader. The DPPH radical scavenging capacity was calculated using a Trolox standard curve and expressed as milligrams of Trolox equivalent (TE) per gram. The experiment was independently repeated three times.

Ferric reducing antioxidant power (FRAP): The FRAP working solution was prepared by mixing sodium acetate buffer (300 mM, pH 3.6), TPTZ solution (10 nM), and FeCl3 solution (20 mM) in a 10:1:1 ratio. Twenty microliters (20 μL) of appropriately diluted sample were mixed with 180 μL of the FRAP working solution. The mixture was allowed to react in the dark at 25 °C for 5 min. The absorbance at 593 nm was measured using a microplate reader. The FRAP was calculated using a Trolox standard curve and expressed as milligrams of TE per gram. The experiment was independently repeated three times.

Oxygen radical absorbance capacity (ORAC): Twenty-five microliters (25 μL) of appropriately diluted sample were added to 150 μL of 40 nM sodium fluorescein solution in a 96-well plate. The mixture was allowed to react at 37 °C in the dark for 10 min. Then, 25 μL of 150 mM AAPH solution was added. Fluorescence intensity was measured at an excitation wavelength of 485 nm and an emission wavelength of 535 nm, with readings taken every 2 min for a total of 2 h. PBS was used as a control, and wells without AAPH served as initial fluorescence readings. The difference in the area under the sodium fluorescein decay curve (net AUC) between the blank and the sample was used for calculation. The net AUC was calculated as follows: Net AUC = AUC_sample_ − AUC_AAPH+_, AUC = 2 × (f_0_ + f_1_ + … + f_n_) − f_n_ − f_0_. The ORAC equivalents were calculated using a Trolox standard curve and expressed as milligrams of TE per gram. The experiment was independently repeated three times.

ABTS radical scavenging activity: A 7 mM ABTS and 2.6 mM K_2_S_2_O_8_ solution were mixed in a 1:1 ratio and allowed to react in the dark at 25 °C for 12 h. The resulting ABTS working solution was diluted with a solvent to obtain an OD_734nm_ ≈ 0.63. Ten microliters (10 μL) of appropriately diluted sample were mixed with 200 μL of the ABTS working solution. The mixture was allowed to react in the dark at 25 °C for 5 min, and the absorbance at 734 nm was measured. The ABTS radical scavenging activity was calculated using a Trolox standard curve and expressed as milligrams of TE per gram. The experiment was independently repeated three times.

Calculation of the antioxidant capacity ratio (%): The antioxidant capacity ratio represents the degree of explanation of the antioxidant capacity of the detected substance to the overall substance. According to the contents of polyphenols and terpenoids in each peel and flesh, 20 kinds of standard substances were compounded according to the average value, and the chemical antioxidation of these compounds, such as DPPH, FRAP, ORAC and ABTS, were evaluated. The ratio of these evaluation values to the values of fruit peel and flesh extracts is the antioxidant capacity ratio. The antioxidant capacity ratio = CAP compound/CAP extract, where CAP is the abbreviation of chemical antioxidant capacity.

### 2.5. Cell Culture and Cell Viability Assay

Mouse liver cell line AML-12 was cultured in DMEM/F12 medium supplemented with 10% fetal bovine serum (FBS) and 1% antibiotic–antifungal mixture (100 U/mL penicillin, 100 μg/mL streptomycin, 0.25 μg/mL amphotericin B). The cells were incubated in an incubator at 37 °C with a CO_2_ concentration of 5%. Passage was carried out when the cell confluence reached approximately 70–80%.

Upon reaching the logarithmic growth phase, AML-12 cells were seeded in a culture plate and incubated in serum-free DMEM/F12 medium for 12 h. Subsequently, the cells were treated with 200 mM ethanol, and 25~400 μg/mL loquat fruit extracts and pure monomers were added for co-incubation to establish an alcohol-induced liver injury cell model. Cell viability was assessed using a CCK-8 kit. After removing the cell culture medium, the cells were washed twice with PBS. The CCK-8 solution was diluted with serum-free DMEM/F12 medium (1:10) and added to the cell culture plate, followed by incubation in the dark for 1 h. The absorbance values at 620 nm and 450 nm were measured using a microplate reader to calculate the cell activity. DMSO was used as the solvent control, and each experiment was performed in triplicate wells, with at least three independent repetitions.

### 2.6. Determination of Cellular ROS Content and Antioxidant Enzyme Activity

The cells co-cultured with ethanol and various loquat peel and flesh extracts/pure compounds were gently washed twice with PBS, and a 10 μM DCFH-DA fluorescent probe, diluted with serum-free DMEM/F12 medium, was introduced into the well. Following a 20 min incubation at 37 °C, the DCFH-DA solution was aspirated, and the cells were subjected to three washes with serum-free medium. Fluorescence intensity was measured using a microplate reader with an excitation wavelength of 488 nm and an emission wavelength of 525 nm. Each experiment was conducted independently three times. The activities of antioxidant enzymes, including superoxide dismutase (SOD), glutathione peroxidase (GPx), and catalase (CAT), were evaluated using commercially available assay kits, following the protocols outlined in the kit instructions.

### 2.7. Animal Modeling Method

Male C57BL/6J mice, aged 6–8 weeks, were procured from the animal center platform of Zhejiang University. Upon arrival at the animal center, the experimental animals underwent a one-week acclimatization period. Following acclimation, the mice were randomly allocated into 14 groups, each consisting of 5 mice, ensuring comparable initial body weights across the groups. The specific groups comprised: a normal control group (Control), an alcohol model group (EtOH), six groups for loquat peel extract intervention (LYQ-peel, DWX-peel, DHP-peel, DYYD-peel, BY-peel, BS-peel), and six groups for loquat flesh intervention (LYQ-flesh, DWX-flesh, DHP-flesh, DYYD-flesh, BY-flesh, BS-flesh). The experimental protocol encompassed two stages: the dietary intervention stage and the acute alcohol-induced liver injury modeling stage. Over the initial two weeks of the dietary intervention stage, the control and model groups received oral administration of pure water, while the loquat extract intervention groups were administered corresponding fruit flesh and peel extracts at a dose of 200 mg·kg^−1^·BW^−1^·day^−1^. In the third week, the acute alcohol-induced liver injury modeling stage commenced. The control group received daily oral doses of pure water. The EtOH model group was administered a gavage of 50% (*v*/*v*) ethanol solution at a dose of 12 mL·kg^−1^·BW^−1^·day^−1^, one hour after the oral administration of pure water. For the loquat extract intervention groups, a dose of 200 mg·kg^−1^·BW^−1^·day^−1^ of the respective fruit extract was first administered, followed by a gavage of 50% (*v*/*v*) ethanol solution at a dose of 12 mL·kg^−1^·BW^−1^·day^−1^, one hour later. Daily observations were made on the mice’s health status, food intake, and drinking water for any anomalies. Mice were fed on a regular schedule, and their weights were recorded every two days. Prior to the experiment’s conclusion, the mice underwent a fasting period. At the endpoint of the experiment, the mice were euthanized, and their serum, liver, kidney, and intestinal contents were promptly collected. Simultaneously, the weights of critical organs were measured. The experiments were conducted following the ethical guidelines outlined by the animal experimentation committee at Zhejiang University. The animal experiment adhered to ethical approval with the code ZJU20220283.

### 2.8. Animal Serum Index Detection Method

Whole blood was aseptically collected from mice and dispensed into 1.5 mL sterile centrifuge tubes, allowing it to settle at 4 °C for 3–5 h. Subsequently, the tubes were subjected to centrifugation at 3000 rpm and 4 °C for 10 min, facilitating the careful transfer of the resulting supernatant to fresh centrifuge tubes. Serum levels of triglycerides (TG), total cholesterol (TC), high-density lipoprotein cholesterol (HDL-C), low-density lipoprotein cholesterol (LDL-C), as well as aspartate aminotransferase (AST) and alanine aminotransferase (ALT), were quantified using commercially available assay kits and a microplate reader.

### 2.9. Animal Liver Index Detection Method

Lipid peroxidation markers such as malondialdehyde (MDA), along with the activities of key antioxidant enzymes including CAT, SOD, and GPx, were quantified utilizing commercially available assay kits in accordance with the manufacturer’s instructions.

### 2.10. Real Time-PCR (RT-PCR)

Total RNA was extracted following the Trizol protocol. Genomic DNA elimination was performed using the PrimeScript™ RT reagent Kit with gDNA Eraser, followed by cDNA synthesis through reverse transcription. For qPCR detection, the SsoFast™ EvaGreen^®^ Supermix kit was employed, with primer sequences detailed in Table S1. Each experiment was undertaken in triplicate. *β-Actin* was utilized as an internal reference, and the 2^−ΔΔCt^ method was employed to calculate relative gene expression levels.

### 2.11. Statistics

Quantitative experimental data concerning loquat phenolics and terpenoids were gathered from a minimum of three replicates and presented as mean ± standard deviation. Statistical analysis was carried out using SPSS 26.0 software. Differences among samples were evaluated through one-way ANOVA, followed by significance determination via the Tukey test (*p* < 0.05). To assess the correlation between individual compounds and antioxidant capacity, the two-tailed significance test of Spearman’s correlation coefficient was applied (*p* < 0.05).

## 3. Results

### 3.1. Identification and Quantification of Loquat Peel and Flesh Extracts

**Figure 2:**
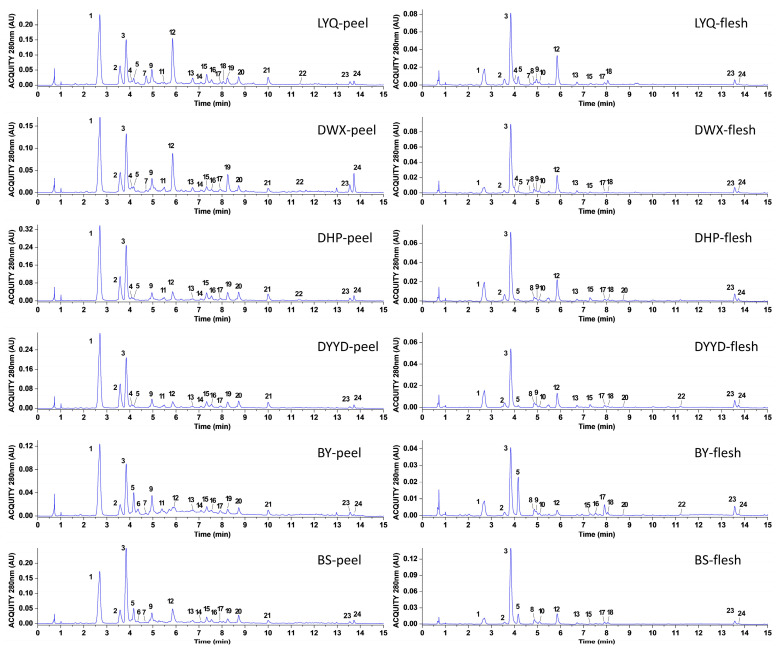
UPLC chromatogram of loquat peel and flesh.

Utilizing UPLC–HRMS, we conducted a comprehensive analysis of compounds present in both the peel and flesh of four red-fleshed loquat varieties and two white-fleshed loquat varieties. Substance identification was accomplished through the integration of retention time, ion fragments, and related data. A total of 24 substances were successfully identified (Figure 2), comprising 22 phenolics and 2 terpenoids (Table 1). Among these, 20 substances, for which standard samples were available, were positively confirmed through data comparison, and their concentrations were quantified employing the standard curve method (Table 2). Notably, among the 24 identified substances, 6 were novel to loquats, specifically: 3-*p*-coumaroylquinic acid, ferulic acid-4-*O*-glucoside, 1-*O*-caffeoylquinic acid, 4-*p*-coumaroylquinic acid, chrysosplenoside B, and kaempferol-3-*O*-(6″-acetyl) glucoside.

The primary polyphenolic compounds in the fruit peel were identified as 5-caffeoylquinic acid and 3-caffeoylquinic acid, while within the fruit flesh, the dominant polyphenolic compound was 3-caffeoylquinic acid. Across all of the loquat fruit varieties, the concentrations of phenolics and terpenoids in the peel consistently surpassed those found in the flesh. Notably, among the 24 identified compounds, 7 exhibited a distinct tissue-specific distribution. Procyanidin B2, procyanidin C1, quercetin-3-*O*-sambubioside, kaempferol-3-*O*-glucoside, and kaempferol-3-*O*-rhamnoside were exclusively present in the fruit peel and absent in the fruit flesh. Conversely, 4-*p*-coumaroylquinic acid and chrysosplenoside B were solely detected in the flesh. Additionally, five compounds, including 4-*O*-caffeoylquinic acid, procyanidin B2, ferulic acid-4-*O*-glucoside, procyanidin C1, and kaempferol-3-*O*-(6″-acetyl)glucoside, demonstrated species specificity. Among these, 4-*O*-caffeoylquinic acid and kaempferol-3-*O*-(6″-acetyl)glucoside were exclusively present in red-fleshed loquats, while procyanidin B2 was confined to the peel of white-fleshed varieties. Terpenoid compounds were detected in all fruit peels and flesh, with the highest levels observed in the ‘DWX’ fruit peel, notably 0.22 ± 0.01 mg/g DW (oleanolic acid) and 0.43 ± 0.02 mg/g DW (ursolic acid).

### 3.2. Chemical Antioxidant Evaluation of Loquat Fruit Extracts

**Figure 3:**
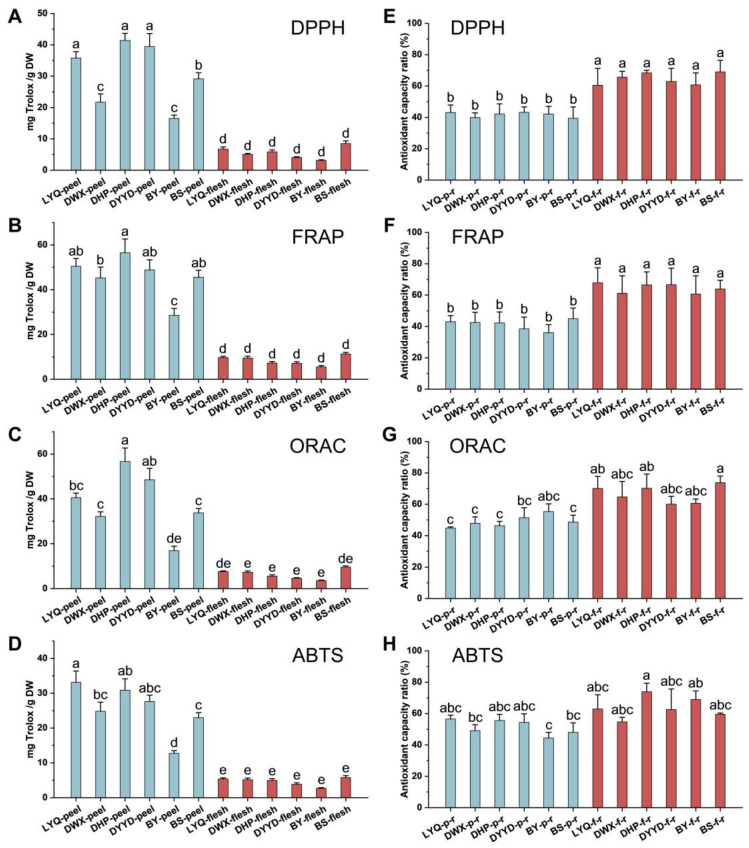
The chemical antioxidant capacity of loquat peel and flesh evaluated by DPPH (**A**), FRAP (**B**), ORAC (**C**), ABTS (**D**) methods and antioxidant capacity ratio of the mixtures recombined with the main detected monomer substances (**E**–**H**). Each value represents three replicates. The different letters on the columns represent significant differences (*p* < 0.05) among different varieties.

Four distinct chemical models, encompassing DPPH, FRAP, ORAC, and ABTS assays, were employed to comprehensively assess the antioxidant potential of both loquat peel and flesh extracts. The results show analogous trends in antioxidant capacity across the four models, as illustrated in Figure 3A–D. Notably, fruit peel extracts showcased notably augmented antioxidant prowess in comparison with their fruit flesh counterparts. This disparity was particularly prominent in the DPPH, FRAP, and ABTS evaluation methodologies, where all fruit peel extracts exhibited markedly superior capabilities in free radical scavenging and metal ion reduction when contrasted with the fruit flesh extracts. Within the cohort of fruit peel extracts, LYQ, DHP, and DYYD varieties displayed heightened antioxidant capacities, while DWX and BY varieties exhibited comparatively subdued antioxidant potentials. Regarding fruit flesh extracts, a marginally elevated antioxidant capacity was discerned in the BS variety, albeit without achieving statistical significance.

The 20 individual compounds that were identified were reconstituted at their respective concentrations present in the fruit peel and flesh extracts for the 4 chemical antioxidant capacity assessments, as depicted in Figure 3E–H. Intriguingly, within the DPPH and FRAP methodologies, the antioxidant capacity proportion of the reconstituted fruit flesh extracts markedly surpassed that of the fruit peel extracts, exceeding a 60% threshold. This outcome implies that the detected phenolics and terpenoid constituents potentially play a more substantial role in driving the antioxidant capacity of the fruit flesh extracts.

Conversely, for the ORAC and ABTS antioxidant evaluation techniques, the antioxidant capacity proportion in the reconstituted fruit peel extracts generally maintained a slightly lower profile compared with that of the reconstituted fruit flesh extracts. However, these variances did not translate into significant overall disparities between the two fractions. Furthermore, within the same tissue, no significant distinctions emerged in the proportion of antioxidant capacity across different varieties of reconstituted extracts.

**Figure 4:**
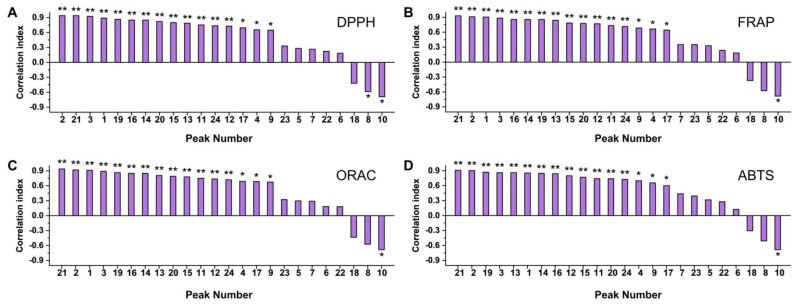
Correlation of the primary detected monomer substances with chemical antioxidant capacity. (**A**) DPPH; (**B**) FRAP; (**C**) ORAC; (**D**) ABTS. The horizontal coordinate is shown as the peak order of the corresponding material: 1: 5-caffeoylquinic acid, 2: 3-*p*-coumaroylquinic acid, 3: 3-caffeoylquinic acid, 4: 4-*O*-caffeoylquinic acid, 5: sinapic acid, 6: procyanidin B2, 7: ferulic acid-4-*O*-glucoside, 8: 1-*O*-caffeoylquinic acid, 9: caffeic acid, 10: 4-*p*-coumaroylquinic acid, 11: procyanidin C1, 12: 5-feruloylquinic acid, 13: quercetin-3-*O*-neohesperidoside, 14: quercetin-3-*O*-sambubioside, 15: quercetin-3-*O*-galactoside, 16: kaempferol-3-*O*-neohesperidoside, 17: 3,6,7,2′,4′-pentamethylquercetagetin 3′-*O*-glucoside, 18: chrysosplenoside B, 19: kaempferol-3-*O*-glucoside, 20: quercetin-3-*O*-rhamnoside, 21: kaempferol-3-*O*-rhamnoside, 22: kaempferol-3-*O*-(6″-acetyl)glucoside, 23: oleanolic acid, and 24: ursolic acid. The * indicates that the substance has a significant positive or negative correlation with the indicated antioxidant capacity, *, *p* < 0.05; **, *p* < 0.01.

The association between antioxidant capacity and content of loquat flesh and peel extracts was explored, yielding the findings illustrated in Figure 4. Within the roster of 24 compounds, 16 exhibited robust positive correlations, with correlation coefficients surpassing 0.6. Interestingly, three compounds manifested inverse correlations with the overall antioxidant capacity; notably, 4-*p*-coumaroylquinic acid demonstrated statistical significance across all assessment methods, with correlation coefficients ranging from −0.683 to −0.690. It is essential to underscore that these correlation coefficients depict the interplay between compound expression patterns and the overall antioxidant capacity. It is crucial to emphasize that negative correlations observed among specific substances did not necessarily indicate their promotion of oxidative stress or possession of pro-oxidant attributes.

### 3.3. In Vitro Antioxidant Evaluation of Loquat Fruit Extracts

**Figure 5:**
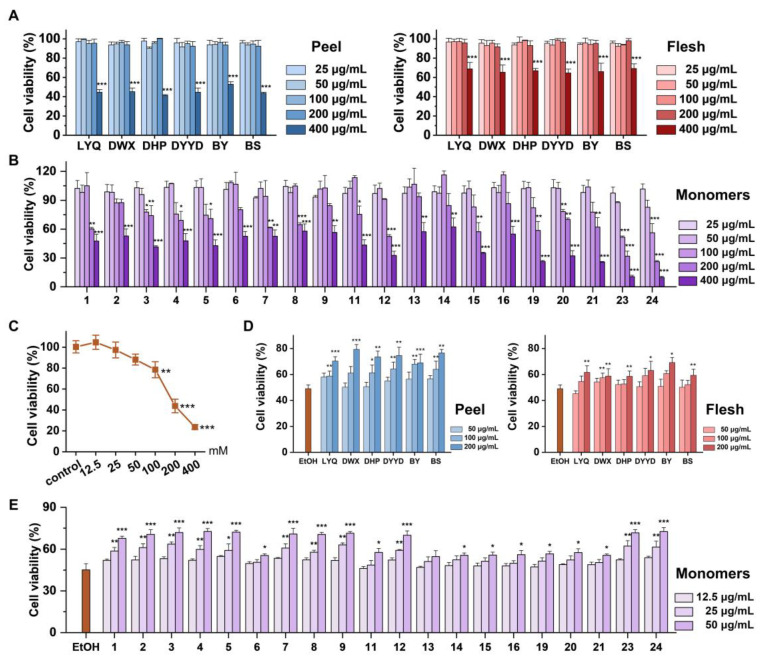
Cell viabilities of AML-12 cells under different loquat peel and flesh extracts and ethanol-induced oxidative stress measured using a cell counting kit-8 assay. (**A**) Effects of loquat peel and flesh extracts on the cell viability of AML12 cells; (**B**) effects of monomers detected in loquat peel and flesh on the cell viability of AML12 cells; (**C**) cell viability of AML-12 cells under different ethanol treatment concentrations; (**D**) effects of loquat extract on the cell viability of AML-12 cells after ethanol modeling; (**E**) effects of monomers detected in loquat peel and flesh on the cell viability of AML-12 cells after ethanol modeling. EtOH, ethanol; the horizontal coordinate is shown as the peak order of the corresponding material: 1: 5-caffeoylquinic acid, 2: 3-*p*-coumaroylquinic acid, 3: 3-caffeoylquinic acid, 4: 4-*O*-caffeoylquinic acid, 5: sinapic acid, 6: procyanidin B2, 7: ferulic acid-4-*O*-glucoside, 8: 1-*O*-caffeoylquinic acid, 9: caffeic acid, 11: procyanidin C1, 12: 5-feruloylquinic acid, 13: quercetin-3-*O*-neohesperidoside, 14: quercetin-3-*O*-sambubioside, 15: quercetin-3-*O*-galactoside, 16: kaempferol-3-*O*-neohesperidoside, 19: kaempferol-3-*O*-glucoside, 20: quercetin-3-*O*-rhamnoside, 21: kaempferol-3-*O*-rhamnoside, 23: oleanolic acid, and 24: ursolic acid; the * indicates significant difference from control group (**A**–**C**) or EtOH group (**D**,**E**), *, *p* < 0.05; **, *p* < 0.01; ***, *p* < 0.001.

The biological effects of loquat fruit peel and flesh extracts, along with the identified individual phenolic and terpenoid compounds, were investigated in vitro using the mouse normal liver cell line AML-12. The viability of these cells was determined through the CCK-8 assay, revealing that concentrations of up to 200 μg/mL for both fruit peel and flesh extracts exhibited no cytotoxicity to AML-12 cells (Figure 5A). Similarly, the assessment of cell viability for the individual phenolic and terpenoid compounds found in loquat fruit demonstrated that no compounds, when present at concentrations below 50 μg/mL, induced cytotoxic effects on AML-12 cells (Figure 5B). To ensure that neither the extracts nor the individual compounds interfered with ethanol-induced cellular damage, subsequent experiments employed treatment concentrations of 200 μg/mL for extracts and 50 μg/mL for individual compounds. Cells were pre-treated with these concentrations for 12 h, followed by co-incubation with various ethanol concentrations for 24 h to induce cellular damage. Viability was then assessed, revealing a significant reduction in cell viability to 78.54% at ethanol concentrations above 100 mM. At an ethanol concentration of 200 mM, cell viability plummeted to around 43.81%. Consequently, an ethanol concentration of 200 mM was selected for the subsequent modeling experiments (Figure 5C).

As depicted in Figure 5D, both loquat fruit flesh and peel extracts exhibited the ability to counteract the ethanol-induced reduction in cell viability. This protective effect displayed a discernible dose-dependent pattern, with notable enhancement observed at the concentration of 200 μg/mL. The results presented in Figure 5E highlight that, among the 20 individual compounds detected in the loquat fruit extracts, 19 demonstrated significant potential in ameliorating the ethanol-induced decline in cell viability. Quercetin-3-*O*-neohesperidoside emerged as the sole exception, displaying no significant impact on cell viability, whether inhibitory or enhancing.

Of particular significance was the observation that compounds with lower molecular weights exhibited a more pronounced enhancement of cell viability. Specifically, compounds such as 5-caffeoylquinic acid, 3-*p*-coumaroylquinic acid, 3-caffeoylquinic acid, 4-*O*-caffeoylquinic acid, sinapic acid, ferulic acid-4-*O*-glucoside, 1-*O*-caffeoylquinic acid, caffeic acid, 5-feruloylquinic acid, oleanolic acid, and ursolic acid displayed remarkably significant effects, leading to cell viabilities ranging from 67.69% to 72.62%.

Conversely, compounds characterized by higher molecular weights, such as procyanidin B2, procyanidin C1, quercetin-3-*O*-sambubioside, quercetin-3-*O*-galactoside, kaempferol-3-*O*-neohesperidoside, kaempferol-3-*O*-glucoside, quercetin-3-*O*-rhamnoside, and kaempferol-3-*O*-rhamnoside, demonstrated noteworthy enhancement at a treatment concentration of 50 μg/mL, resulting in cell viabilities ranging from 55.58% to 57.75%.

**Figure 6:**
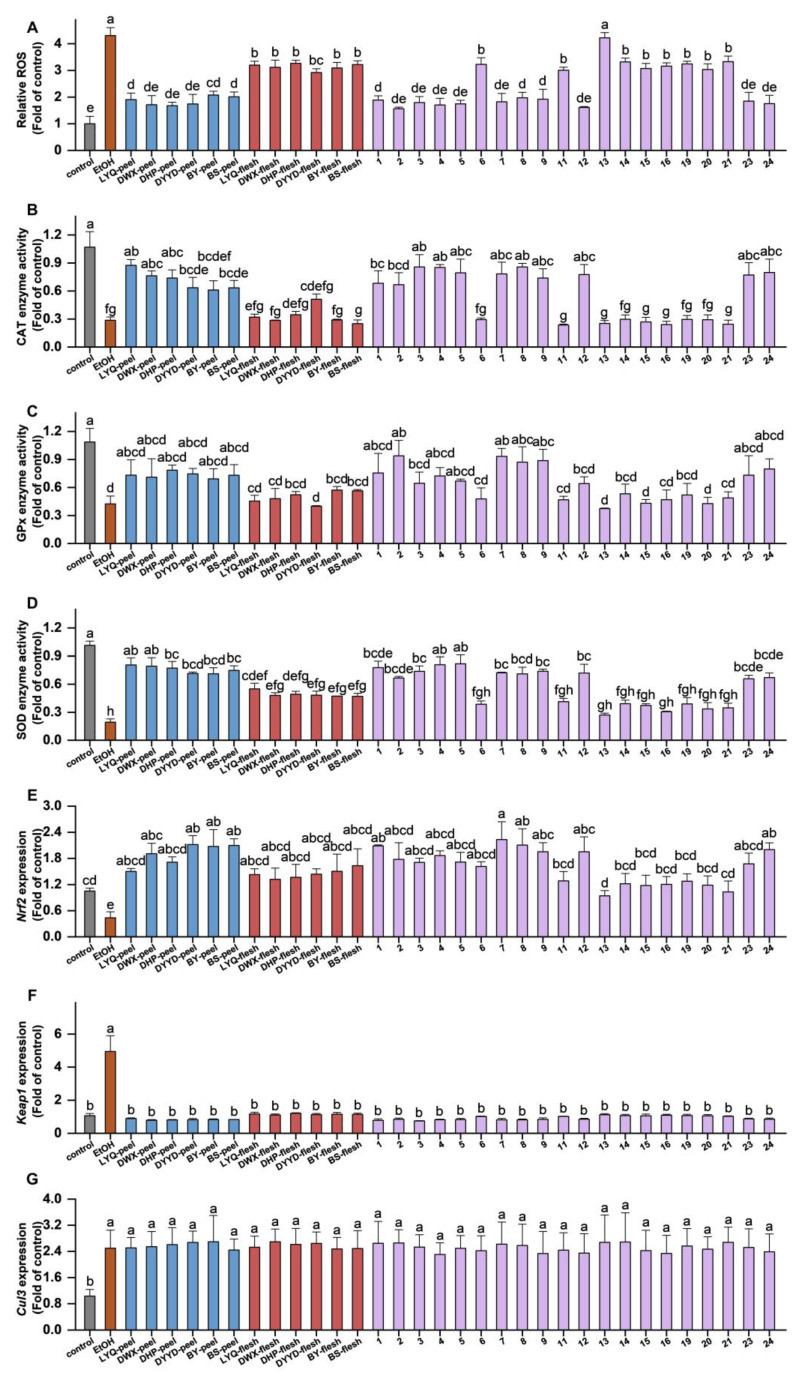
Effects of loquat extracts and monomers on ethanol-induced oxidative stress in AML-12 cells. Effects of loquat extracts and monomers on ROS content in ethanol-induced oxidative stressed AML-12 cells detected by DCFH-DA (**A**); effects of loquat extracts and monomers on CAT (**B**), GPx (**C**), and SOD (**D**) enzyme activities in ethanol-induced oxidative stressed AML-12 cells; effects of loquat extracts and monomers on *Nrf2* (**E**), *Keap1* (**F**), and *Cul3* (**G**) expressions in ethanol-induced oxidative stressed AML-12 cells. EtOH, ethanol; the horizontal coordinate is shown as the peak order of the corresponding material: 1: 5-caffeoylquinic acid, 2: 3-*p*-coumaroylquinic acid, 3: 3-caffeoylquinic acid, 4: 4-*O*-caffeoylquinic acid, 5: sinapic acid, 6: procyanidin B2, 7: ferulic acid-4-*O*-glucoside, 8: 1-*O*-caffeoylquinic acid, 9: caffeic acid, 11: procyanidin C1, 12: 5-feruloylquinic acid, 13: quercetin-3-*O*-neohesperidoside, 14: quercetin-3-*O*-sambubioside, 15: quercetin-3-*O*-galactoside, 16: kaempferol-3-*O*-neohesperidoside, 19: kaempferol-3-*O*-glucoside, 20: quercetin-3-*O*-rhamnoside, 21: kaempferol-3-*O*-rhamnoside, 23: oleanolic acid, and 24: ursolic acid; columns with different letters indicate a significant difference at *p* < 0.05.

The assessment of ROS content in AML-12 cells was conducted using the DCFH-DA probe. As illustrated in Figure 6A, alcohol-induced a substantial accumulation of ROS in AML-12 cells, surpassing fourfold that of the control group. Notably, pretreatment with 200 μg/mL of loquat fruit extract resulted in a pronounced inhibition of ROS accumulation. Both peel and flesh extracts from the six loquat fruit varieties exhibited significant inhibition of ROS content in the cells. It is noteworthy that the peel extract exhibited a stronger ROS inhibition ability compared with the flesh extract, although no substantial variations were observed among different varieties.

Among the 19 individual compounds assessed, all demonstrated significant capacity in inhibiting ROS, except for quercetin-3-*O*-neohesperidoside, which did not exhibit significant modulation of ROS levels. This observation harmoniously aligns with the outcomes of cell viability assays, suggesting a potential relationship between the inhibitory effects of loquat extracts and individual compounds on ethanol-induced AML-12 cell activity and their capacity to restrain ROS accumulation.

Enzyme activities of CAT, GPx, and SOD were evaluated, revealing that alcohol substantially suppressed the activities of all three antioxidant enzymes. However, the influence of loquat fruit extract and individual compounds on these antioxidant enzymes displayed specificity. In terms of CAT enzyme activity (Figure 6B), the fruit peel extract exhibited a notable enhancement, whereas the fruit flesh extract did not display a significant regulatory effect. The impact on CAT enzyme activity was solely dependent on tissue specificity, rather than variety. Among the individual compounds, 11 substances, including 3-caffeoylquinic acid, 4-*O*-caffeoylquinic acid, 1-*O*-caffeoylquinic acid, sinapic acid, ferulic acid-4-*O*-glucoside, caffeic acid, 5-feruloylquinic acid, oleanolic acid, ursolic acid, 5-caffeoylquinic acid, and 3-*p*-coumaroylquinic acid, demonstrated significant induction of CAT enzyme activity, aligning with their ROS inhibition capacity. Regarding GPx enzyme (Figure 6C), none of the loquat extracts exhibited significant modulation of enzyme activity. Among the individual compounds, only 3-*p*-coumaroylquinic acid, ferulic acid-4-*O*-glucoside, 1-*O*-caffeoylquinic acid, and caffeic acid displayed enhanced GPx enzyme activity, while the remaining compounds did not exhibit substantial regulatory effects. For SOD enzyme (Figure 6D), both loquat fruit peel and flesh extracts led to an increase in SOD enzyme activity, with no significant variations among the different varieties. Among the individual compounds, 11 substances demonstrated regulatory effects on SOD enzyme activity, mirroring the 11 compounds that displayed CAT enzyme regulatory activity. In summary, the findings suggest a stronger correlation between the antioxidant regulatory activity of loquat fruit extract and the modulation of CAT and SOD enzyme activities. Additionally, the regulatory capacity of individual compounds might be associated with their molecular weight.

The *Nrf2-Keap1-Cul3* pathway stands as a pivotal cellular antioxidant regulatory mechanism. Through RT-PCR analysis of the gene expressions, it was revealed that alcohol notably suppressed *Nrf2* expression while inducing the expression of *Keap1* and *Cul3*. This observation implies that alcohol potentially hampers *Nrf2* activation and expression by promoting *Keap1* and *Cul3* expression, thereby curbing both antioxidant enzyme activity and ROS clearance within cells.

Figure 6E demonstrates the induction effect of loquat fruit extracts on *Nrf2* expression. All fruit extracts and individual compounds displayed significant *Nrf2* expression induction. In terms of *Keap1* (Figure 6F), both loquat fruit extracts and individual compounds exhibited noteworthy inhibitory effects on *Keap1* expression. However, concerning *Cul3* (Figure 6G), neither the extracts nor the individual compounds displayed a significant regulatory effect. The outcomes suggest that the impacts of loquat extracts and individual compounds on the antioxidant regulatory pathway likely encompass a comprehensive modulation of *Nrf2* and *Keap1* expression, with a relatively weaker correlation to *Cul3* expression.

### 3.4. In Vivo Antioxidant Evaluation of Loquat Fruit Extracts

**Figure 7:**
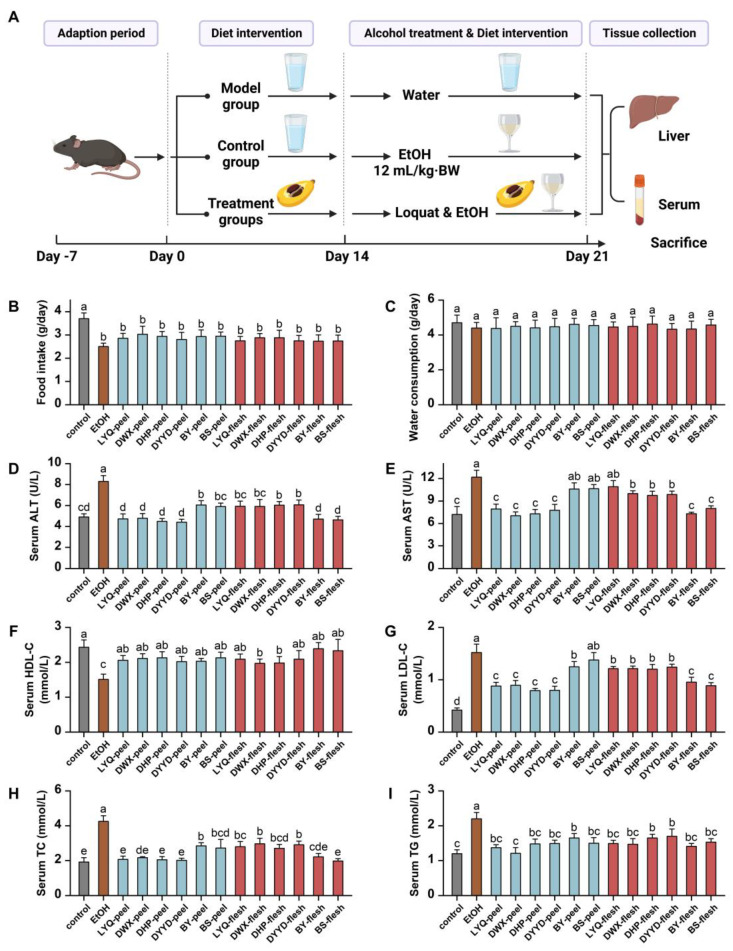
Effects of loquat extracts on serum markers of C57/BL6 mice fed with alcohol. (**A**) Experimental schematic; (**B**) average food intake amount; (**C**) water consumption amount; (**D**) serum alanine aminotransferase (ALT) activity; (**E**) serum aspartate aminotransferase (AST) activity; (**F**) serum high-density lipoprotein cholesterol (HDL-C) level; (**G**) serum low-density lipoprotein cholesterol (LDL-C) level; (**H**) serum total cholesterol (TC) level; (**I**) serum triglyceride (TG) levels. EtOH, ethanol; BW, body weight; columns with different letters indicate significant difference at *p* < 0.05.

This study delved into the hepatoprotective potentials of loquat fruit extract utilizing an alcohol-induced C57/BL6 mouse model (Figure 7A). The outcomes revealed that gastric gavage administration of alcohol substantially curtailed the average food intake of the mice (Figure 7B), although it did not markedly impact water consumption (Figure 7C). Notably, alcohol exposure prompted a decline in the body weight of the mice, with loquat fruit extract consumption moderately mitigating this effect, though the variance was not statistically significant (Figure S1).

Alanine aminotransferase (ALT) and aspartate aminotransferase (AST) are crucial markers of liver health. As illustrated in Figure 7D,E, alcohol ingestion prompted a notable increase in ALT and AST enzyme activities, signifying liver impairment. However, the introduction of loquat fruit extract exerted an inhibitory influence on the aberrant rise of ALT and AST enzyme activities induced by alcohol. Concerning ALT enzyme, both loquat fruit peel and flesh extracts significantly counteracted the alcohol-induced effect. In the case of AST enzyme, eight groups of loquat fruit extracts (comprising four fruit peel extracts and four fruit flesh extracts) exhibited significant inhibition of AST enzyme activity. While the remaining three groups exhibited slightly lower AST enzyme activity in comparison with the alcohol-induced group, the distinction was not statistically significant. These findings suggest that loquat fruit extract holds a protective potential for the liver by mitigating the irregular elevation of ALT and AST enzyme activities triggered by alcohol consumption.

The liver stands as a pivotal metabolic organ, particularly in the context of lipid metabolism. As depicted in Figure 7F–I, alcohol distinctly subdued the expression of high-density lipoprotein cholesterol (HDL-C) and concurrently escalated the levels of low-density lipoprotein cholesterol (LDL-C), total cholesterol (TC), and triglycerides (TG). These findings underscore the disruption of blood lipid metabolism induced by alcohol administration, further accentuating the liver impairment inherent in the alcohol-induced model. Remarkably, loquat fruit extract exhibited a marked enhancement in HDL-C expression. All fruit peel extracts, alongside four fruit flesh extracts, reached a comparable level as the control group, while the remaining two groups also demonstrated significant enhancement in comparison with the model group. In relation to LDL-C, loquat fruit flesh extracts notably curbed the alcohol-induced elevation of this lipid marker. Among the fruit peel extracts, it was solely the BS-peel group that did not achieve a significant reduction, while the remaining five groups exhibited a pronounced reduction in the alcohol-induced elevation. In the context of TC and TG, every treatment group significantly repressed the alcohol-induced overexpression of these markers, with the majority of treatment groups restoring TC and TG levels to a normal range.

**Figure 8:**
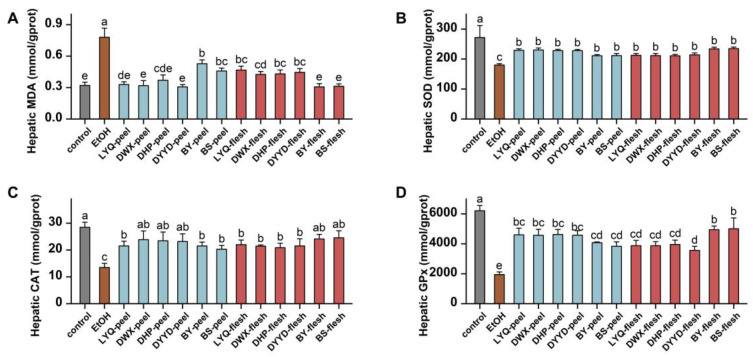
Effects of loquat extracts on liver oxidative stress status and antioxidant enzymes activities of C57/BL6 mice fed with alcohol. (**A**) Hepatic malondialdehyde (MDA) content; (**B**) hepatic liver superoxide dismutase (SOD) activity; (**C**) liver catalase (CAT) activity; (**D**) liver glutathione peroxidase (GPx) activity. EtOH, ethanol; columns with different letters indicate significant difference at *p* < 0.05.

The evaluation of lipid peroxidation levels and antioxidant enzyme activities within the liver uncovered the substantial induction of MDA accumulation by alcohol (Figure 8A), coupled with a marked inhibition of liver SOD, CAT, and GPx antioxidant enzyme activities (Figure 8B–D). These indicators unequivocally reflect the disturbance in oxidative stress equilibrium triggered by alcohol within the liver. Significantly, both loquat fruit peel and flesh extracts effectively curbed the rise of MDA triggered by alcohol, underscoring their capacity to impede lipid peroxidation. Notably, in the case of CAT, SOD, and GPx enzymes, loquat extracts demonstrated remarkable efficacy in mitigating the inhibitory impact of alcohol on enzyme activities. This compellingly illustrates that loquat extracts play a substantial role in counteracting the alcohol-induced suppression of key antioxidant enzyme activities, namely CAT, SOD, and GPx. These findings collectively attest to the formidable liver-protective effects of loquat fruit extract against alcohol-induced oxidative stress, characterized by the inhibition of lipid peroxidation and the restoration of pivotal antioxidant enzyme activities. Such findings suggest that loquat fruit extract holds promising potential in attenuating oxidative damage and fostering liver well-being in the milieu of alcohol-induced stress.

## 4. Discussion

In this study, we successfully identified and quantified a total of 24 substances in loquat fruit using UPLC-HRMS, comprising 22 phenolics and 2 terpenoids. These findings underscore the diversity and complexity of the chemical composition of loquat fruit. Among these 24 substances, 6 compounds were unveiled in loquat for the first time, unveiling a wealth of previously undiscovered plant chemical diversity within this fruit. This revelation underscores its significant contribution to the broader realm of botanical chemical knowledge. Prior research has also hinted at the presence of diverse phenolic acid compounds, notably caffeic acid derivatives, in loquat fruit [21]. These substances are also present in other parts of the loquat plant, such as leaves and flowers, which have traditionally been employed for medicinal purposes [2,3,22], suggesting potential analogous effects of loquat fruit. In the realm of flavonoid identification, our investigation revealed that loquat’s flavonoids primarily encompass glycoside derivatives of kaempferol and quercetin. This particular pairing of aglycone and glycoside is a recurrent theme in Rosaceae fruits such as apples [23], peaches [24], pears [25], and others, all of which manifest as flavanol derivatives. Intriguingly, we also pinpointed a polymethoxylated flavonoid, specifically 3,6,7,2′,4′-pentamethylquercetagetin 3′-*O*-glucoside, within loquat fruit. This type of polymethoxylated flavonoid, marked by more than four methoxy groups, was previously believed to be prevalent mainly in the peels and leaves of citrus fruits from the Rutaceae family [26]. This marks the inaugural identification of such a compound within a Rosaceae fruit, as exemplified by the loquat.

Utilizing both qualitative and quantitative analyses, our investigation encompassed two key objectives. Firstly, we aimed to identify the principal compounds within each loquat sample, and secondly, we sought to comprehend the distribution patterns of these compounds across various attributes, including tissue sections and distinct varieties. The research outcomes unveiled intriguing insights into the composition of the 24 identified compounds. Remarkably, nine of these compounds were found to be uniformly distributed across all tissue parts of the loquat. Among these, 5-caffeoylquinic acid and 3-caffeoylquinic acid emerged as predominant components within the fruit peel, while 3-caffeoylquinic acid took precedence in the fruit flesh.

Evidence from studies on other Rosaceae fruits, such as peaches, apples, and hawthorns, have illuminated the relationship between compound content and various attributes, encompassing color [27], functionality [28], resistance [29], and flavor [30]. Notably, in this context, our investigation ventured beyond the traditional focus on flower, leaf, fruit, and seed content variations in loquat [2,4,12,19,30]. We delved into uncharted territory, scrutinizing the content disparities within the consumable components of loquat, namely the fruit’s peel and flesh. Interestingly, it is widely acknowledged that bioactive substances tend to be more concentrated in the inedible sections of fruits. However, our study revealed the presence of certain compounds, such as 4-*p*-coumaroylquinic acid and chrysosplenoside B, solely within the fruit flesh. This not only underscores the tissue-specific synthesis of natural compounds but also underscores the fruit flesh’s distinct status as a reservoir of these unique bioactive entities.

Alcohol consumption stands as a notable instigator of liver damage arising from dietary sources, with oxidative stress constituting a primary mechanism through which alcohol inflicts harm upon the liver. The multifaceted pathways through which alcohol triggers oxidative damage encompass the generation of oxygen radicals such as superoxide anions (O2^·−^) and hydroxyl radicals (·OH) via enzymatic processes facilitated by alcohol dehydrogenase (ADH) and aldehyde dehydrogenase (ALDH) during alcohol metabolism [31]. This cascade induces lipid peroxidation, culminating in oxidative harm to cell membranes [31], while concurrently disrupting the innate antioxidant defense mechanisms within cells, including key antioxidant enzymes such as SOD, GPx, and CAT [32]. Consequently, the assessment of the potential of natural compounds in alleviating alcohol-induced oxidative stress mandates the consideration of diverse facets, ranging from their capacity for free radical scavenging to their modulation of peroxidase activity and the attenuation of liver lipid peroxidation. In the ambit of this study, our methodology encompassed a gamut of approaches. Employing chemical antioxidant models such as DPPH, FRAP, ORAC, and ABTS, we elucidated the free radical scavenging and metal ion reduction capabilities inherent in loquat extracts. To delve deeper into the mechanisms, we leveraged an in vitro antioxidant model founded upon the AML-12 normal mouse liver cell line, thereby gauging the prowess of the extracts in inhibiting alcohol-induced cell proliferation while influencing the antioxidant enzyme system. Augmenting our understanding, we undertook a comprehensive assessment of the ameliorative potential of loquat extract in the context of alcohol-induced liver damage, employing C57/BL6 mice as an animal model for in vivo investigations.

During the chemical antioxidant assessment, our research outcomes have illuminated free radical scavenging and metal ion reduction capabilities, underscored by discernible tissue and variety distinctions. On the whole, the peel exhibited a more potent antioxidant capacity than the flesh. Within the flesh, the ‘BS’ variety notably displayed slightly elevated antioxidant prowess. Notably lauded for its exceptional flavor profile [33,34], we regard the ‘BS’ variety as a remarkable exemplar that marries both taste and health-enhancing attributes. Nevertheless, the discernible free radical scavenging abilities exhibited by loquats underscore their potential in counteracting alcohol-triggered oxidative stress. To ascertain the contribution of the identified compounds within loquats to this antioxidant efficacy, we employed two distinct strategies.

First, we amalgamated the detected compounds based on their concentrations and gauged their resultant antioxidant capacity. This evaluation facilitated the computation of reduction ratios by contrasting this composite capacity with the antioxidant potential of the corresponding extract. Second, we delved into the correlation between the calculated compound content within the extract and its attendant antioxidant capacity. These reduction ratios have unveiled the way in which the peel’s reduction capacity spans a spectrum of 40% to 60%, while the flesh’s reduction spans a broader range of 60% to 80%. Significantly, within the DPPH and FRAP evaluations, the flesh’s reduction ratio notably eclipsed that of the peel, suggesting the possible presence of compounds not captured by this specific assay, potentially more abundant in the peel. This variance might also be attributed to synergistic interactions amongst compounds arising from their structural attributes, fostering an antioxidant synergy greater than that of compound blends and echoing observations in prior studies [35,36].

Our adoption of correlation analysis serves as an illuminating avenue by which to unearth active constituents within compound assemblages. In this investigation, correlation analysis has underscored the intrinsic link between compound expression levels within extracts and their overarching antioxidant capacity. Remarkably, robust positive correlations between the content of 16 compounds and the extracts’ antioxidant capabilities have come to light. It is pertinent to note that the content of 4-*p*-coumaroylquinic acid exhibited a consistent negative correlation across all antioxidant evaluation methods. Similarly, 1-*O*-caffeoylquinic acid demonstrated a negative correlation coefficient in the context of the DPPH evaluation. Subsequent cellular experiments uncovered 1-*O*-caffeoylquinic acid’s capacity for cellular ROS clearance. This intriguing phenomenon might be attributed to an expression pattern counter to the majority of other antioxidative compounds exhibiting chemical antioxidant capabilities. In the case of 4-*p*-coumaroylquinic acid, the unavailability of standards precluded a direct validation of its free radical scavenging efficacy. However, it is prudent to highlight that certain compounds could manifest divergent antioxidant capacities when examined across distinct chemical, cellular, or animal models [37]. In this vein, the potential exists for 4-*p*-coumaroylquinic acid to demonstrate a comparable effect, potentially activating the cell’s inherent antioxidant pathways to manifest its antioxidative role—a facet warranting rigorous further investigation.

A significant mechanism underlying alcohol-induced cell damage involves the disruption of the cell’s innate antioxidant defense system. Our investigation has illuminated the way in which loquat possesses the ability to activate antioxidant enzymes through the Nrf2-Keap1 pathway, effectively curbing ROS accumulation and safeguarding liver cells. The Nrf2-Keap1-Cul3 pathway plays a pivotal role in cellular responses to oxidative stress and toxic agents. This intricate cascade orchestrates Nrf2 stability and activity, governing the modulation of antioxidant and cell-protective gene expression within the cellular nucleus [37].

The triumvirate of key participants within this pathway includes nuclear factor erythroid 2-related factor 2 (Nrf2), a transcription factor that dictates the expression of antioxidant and cell-protective genes based on its activity level; Kelch-like ECH-associated protein 1 (Keap1), a negative regulator of Nrf2 that retains Nrf2 in the cytoplasm and orchestrates its degradation, thereby controlling Nrf2 activity; and Cullin 3 (Cul3), a structural protein that collaborates with other molecules to form a Cullin–RING E3 ligase complex (CRL3), critical in Nrf2 degradation [38]. Under normal conditions, Keap1 binds with Nrf2, provoking Nrf2 oligomerization and targeted degradation. However, when cells encounter triggers such as oxidative stress, ionizing radiation, or toxic compounds, Keap1 undergoes conformational shifts that hinder its effective capture and degradation of Nrf2 [11]. This renders Nrf2 free from Keap1 control, enabling its nuclear translocation. Inside the nucleus, Nrf2 partners with antioxidant response elements (ARE), instigating the transcription of an array of cell-protective genes encompassing antioxidant enzymes, heat shock proteins, and oxidoreductases [39]. This concerted action by the proteins synthesized from these genes collaborates to mitigate cellular damage and counteract oxidative stress.

In instances of alcoholic liver injury, alcohol ingestion amplifies cellular oxidative stress, inciting the generation of an abundance of free radicals and oxidative byproducts, thus setting off inflammation and damage within liver tissue. Our study has illuminated how alcohol treatment, under specific concentrations and durations, may induce the upregulation of *Keap1* expression while suppressing *Nrf2* expression, thereby disturbing the equilibrium of the antioxidant enzyme system. However, the administration of loquat extract and its constituents holds the potential to revert this process, effectively reinstating the protective influence of *Nrf2* and reinstating cellular antioxidant mechanisms.

The cellular antioxidant enzyme system constitutes a cohort of enzymes within cells that serve as a frontline defense against the detrimental impact of free radicals and oxidative molecules generated by oxidative stress. These enzymes assume a protective role by neutralizing free radicals, rehabilitating oxidized compounds, and upholding the cellular redox equilibrium. Their collective action ensures cell safeguarding against oxidative stress. In the context of alcoholic liver injury, the escalation of oxidative stress due to alcohol consumption presents a formidable challenge to the cellular antioxidant enzyme system. For instance, during alcohol metabolism, an increase in superoxide radicals occurs. Diminished superoxide dismutase (SOD) activity leads to the accumulation of superoxide radicals, intensifying oxidative stress-induced cell damage [31]. Alcohol intake reduces glutathione levels—a coenzyme and substrate for glutathione peroxidase (GPx)—potentially leading to lowered GPx activity and hampered resistance to oxidative stress [40]. Alcohol metabolism augments intracellular hydrogen peroxide production, heightening oxidative stress levels [41]. This underscores the crucial role of catalase (CAT), which decomposes hydrogen peroxide into harmless water and oxygen, effectively mitigating the risk of cellular damage due to hydrogen peroxide oxidation. These antioxidant enzymes—SOD, GPx, CAT—hold pivotal roles in countering alcoholic liver injury, aiding cells in converting detrimental free radicals into innocuous water and oxygen through hydrogen peroxide mediation.

Augmenting the activity of antioxidant enzymes through dietary interventions represents a beneficial strategy to bolster cellular antioxidant defense mechanisms, thereby alleviating alcohol-induced liver damage. Prior investigations have indicated that compounds, such as EGCG, vitamin E, and betaine, could ameliorate liver damage by enhancing antioxidant enzyme activity [42]. Our previous study also illuminated how citrus peel extract has the potential to elevate the expression and functionality of enzymes such as CAT and NQO1, thereby boosting the antioxidant capacity of liver cells [37]. Notably, loquat extract has been unveiled to enhance the functionality of key antioxidant enzymes such as SOD, CAT, and GPx [43,44]. Nevertheless, our research stands as the first to unveil its potent antioxidant capacity against alcohol-induced oxidative stress.

Both chemical and cellular models have corroborated the potential effectiveness of loquat extract in mitigating alcohol-induced oxidative stress. However, validation through oral administration in animal models is a crucial step. Various models exist for simulating alcohol-induced oxidative stress, including the Lieber–DeCarli liquid diet model [45], Tsukamoto–French intragastric feeding (TF), alcohol-preferring rodents (selectively bred), voluntary drinking models, alcohol injection models, acute alcohol models, and mixed models [46]. The acute alcohol model stands out for its ability to rapidly simulate acute effects, simplify experimental complexities, and offer precise control over dosage and timing, making it particularly suitable for the efficient assessment of damage with fruit extracts.

In line with this model, we evaluated serum markers such as ALT, AST, HDL-C, LDL-C, TC, and TG—commonly used biochemical indicators. These markers offer essential insights into the assessment and monitoring of liver damage when investigating alcohol-induced liver injury. Alanine aminotransferase (ALT) and aspartate aminotransferase (AST) serve as indicators of liver function as they are released into the bloodstream when liver cells undergo damage. In cases of alcoholic liver injury, alcohol metabolism induces liver cell damage, resulting in heightened serum ALT and AST levels [47]. Elevated levels of these markers can signify the presence of alcoholic liver injury. High-density lipoprotein cholesterol (HDL-C), known as “good” cholesterol, holds significant relevance for cardiovascular health. During alcoholic liver injury, alcohol’s impact on lipid metabolism might lead to a reduction in HDL-C levels. This reduction could be linked to conditions such as alcoholic fatty liver [48]. Conversely, low-density lipoprotein cholesterol (LDL-C), dubbed “bad” cholesterol, raises the risk of cardiovascular diseases at elevated levels. Alcohol intake has the potential to increase LDL-C levels, heightening the risk of cardiovascular issues [49]. Total cholesterol (TC) and triglycerides (TG) provide insights into blood lipid metabolism. Alcohol consumption might lead to their elevation, reflecting disrupted lipid metabolism, a hallmark of alcoholic liver injury. Evaluating TC and TG levels aids in gauging the extent of liver damage [50]. In essence, these markers offer a comprehensive view of liver function, lipid metabolism, and cellular damage, enabling the evaluation and monitoring of liver health. Our study revealed how alcohol modeling impacts these liver health indicators and unveiled the positive regulatory effect of loquat extract. Collectively, this underscores the potential of loquat as a beneficial dietary resource for mitigating alcohol-induced liver injury.

Lipid peroxidation stands as a significant contributor to the damage inflicted on the liver by alcohol. In the context of alcoholic liver injury, the metabolism of alcohol gives rise to an excessive amount of free radicals and oxidants, which can precipitate an upsurge in lipid peroxidation reactions. This, in turn, fosters the production of malondialdehyde (MDA) [51]. In effect, gauging MDA levels becomes pivotal in gauging the extent of damage, assessing cell membrane impairment, and gauging the severity of oxidative stress. In light of this, our study has brought to light how the oral administration of loquat extract holds the potential to curtail the aberrant elevation of MDA in the liver caused by alcohol. Moreover, the indicators pertaining to the activity of antioxidant enzymes, including SOD, CAT, and GPx, provide further substantiation of the role played by orally administered loquat extract in safeguarding the liver against the detrimental effects triggered by alcohol-induced damage.

## 5. Conclusions

In conclusion, this study conducted a comprehensive investigation into the antioxidant potential and hepatoprotective effects of loquat (*Eriobotrya japonica*) fruit extracts. Through advanced analytical techniques, a total of 24 bioactive compounds, comprising 22 phenolics and 2 terpenoids, were successfully identified and quantified in both fruit peel and flesh extracts. Notably, six novel compounds were discovered, expanding our understanding of loquat’s phytochemical composition. The study’s rigorous chemical antioxidant evaluations using multiple models revealed the remarkable antioxidant capacity of loquat fruit extracts. Fruit peel extracts exhibited heightened antioxidative potential, particularly in free radical scavenging and metal ion reduction, compared with flesh extracts. This disparity was attributed to the distinctive molecular profiles of the two tissues. Intriguingly, the interplay of compounds with varying molecular weights underscored the complexity of antioxidant mechanisms. In vitro assays utilizing a normal liver cell line (AML-12) demonstrated the protective effects of loquat extracts against ethanol-induced oxidative stress. The extracts enhanced cell viability, inhibited ROS accumulation, and modulated antioxidant enzyme activities, primarily catalase (CAT) and superoxide dismutase (SOD). Furthermore, individual compounds within the extracts displayed differential effects on cell viability, reflecting their molecular attributes. The in vivo assessment utilizing an alcohol-induced mouse model reaffirmed the hepatoprotective potential of loquat fruit extracts. The extracts mitigated alcohol-induced disruptions in food intake, body weight, and liver enzyme activities, notably alanine aminotransferase (ALT) and aspartate aminotransferase (AST). Moreover, the extracts effectively restored disturbed blood lipid profiles, contributing to overall liver health. These findings collectively highlight the therapeutic promise of loquat fruit extracts as functional ingredients with hepatoprotective and antioxidant properties. The study not only advances our knowledge of loquat’s phytochemical composition but also suggests its potential application in ameliorating oxidative stress-induced liver damage. Further exploration of the underlying molecular mechanisms and clinical trials will deepen our understanding and unlock novel strategies for combating oxidative stress-related disorders. Thus, loquat emerges as a promising candidate for promoting liver health and overall well-being, making it a subject of considerable interest for future research and therapeutic development.

## Figures and Tables

**Table 1:** Phenolics and terpenoids from those identified in loquat peel and flesh.

Peak No.	TR (min)	(M − H) (*m*/*z*)	(M + H) (*m*/*z*)	Fragment Ions (*m*/*z*)	Formula	Tentative Compounds	References
1	2.6840		355.1027	163.0399, 145.0290, 135.0447, 117.0346	C_16_H_18_O_9_	5-caffeoylquinic acid	[4]
2	3.5737	337.0931		191.0549, 163.0395, 119.0504	C_16_H_18_O_8_	3-*p*-coumaroylquinic acid	[4]
3	3.8384	353.0873		191.0556	C_16_H_18_O_9_	3-caffeoylquinic acid	[4]
4	4.0717	353.0875		191.0552, 179.0337, 173.0446, 135.0447	C_16_H_18_O_9_	4-*O*-caffeoylquinic acid	[12]
5	4.1621		225.0759	175.0392, 147.0449, 91.0566	C_11_H_12_O_5_	sinapic acid	[13]
6	4.3463	577.1331		407.0777, 289.0708, 125.0239	C_30_H_26_O_12_	procyanidin B2	[14]
7	4.7120	355.1030		217.0500. 193.0500, 175.0402, 160.0165, 134.0373, 132.0218	C_16_H_20_O_9_	ferulic acid-4-*O*-glucoside	[15]
8	4.8687	353.0875		191.0550	C_16_H_18_O_9_	1-*O*-caffeoylquinic acid	[16]
9	4.9564		181.0495	163.0394, 145.0299, 135.0444, 117.0348, 89.0409	C_9_H_8_O_4_	caffeic acid	[12]
10	5.0809	337.0927		191.0543, 173.0439, 163.0383, 119.0495, 93.0349	C_16_H_18_O_8_	4-*p*-coumaroylquinic acid	[4]
11	5.4931	865.1978		713.1607, 695.1464, 577.1390, 407.0776, 289.0698, 125.0233	C_45_H_38_O_18_	procyanidin C1	[14]
12	5.8568	367.1034		191.0552, 134.0367, 93.0356	C_17_H_20_O_9_	5-feruloylquinic acid	[4]
13	6.7237		611.1605	303.0507, 287.0557	C_27_H_30_O_16_	quercetin-3-*O*-neohesperidoside	[4]
14	7.0836	595.1289		301.0351, 300.0275, 271.0234	C_26_H_28_O_16_	quercetin-3-*O*-sambubioside	[4]
15	7.3362		465.1026	303.0500	C_21_H_20_O_12_	quercetin-3-*O*-galactoside	[4]
16	7.5455	594.1531		284.0330, 255.0291, 227.0341	C_27_H_30_O_15_	kaempferol-3-*O*-neohesperidoside	[4]
17	7.9160	565.1557		341.0878, 327.0721, 223.0599, 197.0446, 183.0288	C_26_H_30_O_14_	3,6,7,2′,4′-pentamethylquercetagetin 3′-*O*-glucoside	[17]
18	8.0549	535.1444		311.0770, 297.0603, 223.0601, 208.0360, 167.0339	C_25_H_28_O_13_	chrysosplenoside B	[18]
19	8.2381	447.0921		285.0397, 284.0326, 255.0296, 227.0344	C_21_H_20_O_11_	kaempferol-3-*O*-glucoside	[19]
20	8.7253	447.0920		301.0349, 271.0239, 151.0028	C_21_H_20_O_11_	quercetin-3-*O*-rhamnoside	[4]
21	10.0067	431.0976		285.0400, 255.0297, 227.0343	C_21_H_20_O_10_	kaempferol-3-*O*-rhamnoside	[4]
22	11.3885	489.1024		301.0340, 300.0267, 271.0215	C_23_H_22_O_12_	kaempferol-3-*O*-(6″-acetyl)glucoside	[20]
23	13.5589	455.3489		408.3362, 407.3301	C_30_H_48_O_3_	oleanolic acid	[2]
24	13.7329		457.3675	397.2401, 355.1902, 333.1325, 317.1980, 275.1283	C_30_H_48_O_3_	ursolic acid	[2]

**Table 2:** Quantification of phenolics and terpenoids from loquat peel and flesh (mg/g DW).

Peak No.	Tentative Compounds	LYQ-Peel	LYQ-Flesh	DWX-Peel	DWX-Flesh	DHP-Peel	DHP-Flesh	DYYD-Peel	DYYD-Flesh	BY-Peel	BY-Flesh	BS-Peel	BS-Flesh
1	5-caffeoylquinic acid	4.62 ± 0.32 ^c^	0.40 ± 0.00 ^f^	3.59 ± 0.14 ^d^	0.13 ± 0.03 ^f^	7.10 ± 0.01 ^a^	0.40 ± 0.02 ^f^	6.44 ± 0.07 ^b^	0.35 ± 0.00 ^f^	2.73 ± 0.01 ^e^	0.22 ± 0.00 ^f^	3.74 ± 0.03 ^d^	0.26 ± 0.01 ^f^
2	3-*p*-coumaroylquinic acid	1.18 ± 0.11 ^c^	0.12 ± 0.00 ^f^	0.88 ± 0.04 ^d^	0.06 ± 0.00 ^f^	2.01 ± 0.00 ^a^	0.11 ± 0.01 ^f^	1.83 ± 0.00 ^b^	0.08 ± 0.00 ^f^	0.35 ± 0.00 ^e^	0.03 ± 0.01 ^f^	0.84 ± 0.01 ^d^	0.06 ± 0.00 ^f^
3	3-caffeoylquinic acid	2.31 ± 0.16 ^c^	1.38 ± 0.02 ^d^	2.20 ± 0.08 ^c^	1.45 ± 0.04 ^d^	4.15 ± 0.02 ^a^	1.12 ± 0.06 ^e^	3.43 ± 0.03 ^b^	0.90 ± 0.01 ^ef^	1.44 ± 0.01 ^d^	0.70 ± 0.00 ^f^	4.28 ± 0.04 ^a^	2.32 ± 0.07 ^c^
4	4-*O*-caffeoylquinic acid	0.18 ± 0.01 ^b^	0.01 ± 0.00 ^d^	0.17 ± 0.01 ^b^	0.10 ± 0.00 ^c^	0.20 ± 0.00 ^a^	n.d.	0.21 ± 0.00 ^a^	n.d.	n.d.	n.d.	n.d.	n.d.
5	sinapic acid	0.34 ± 0.04 ^c^	0.13 ± 0.00 ^ef^	0.17 ± 0.01 ^e^	0.04 ± 0.00 ^g^	0.15 ± 0.00 ^e^	0.02 ± 0.00 ^g^	0.09 ± 0.02 ^f^	0.03 ± 0.00 ^g^	0.51 ± 0.00 ^b^	0.31 ± 0.00 ^c^	0.67 ± 0.01 ^a^	0.24 ± 0.01 ^d^
6	procyanidin B2	n.d.	n.d.	n.d.	n.d.	n.d.	n.d.	n.d.	n.d.	0.20 ± 0.00 ^a^	n.d.	0.12 ± 0.00 ^b^	n.d.
7	ferulic acid-4-*O*-glucoside	0.39 ± 0.02 ^a^	0.01 ± 0.01 ^b^	0.06 ± 0.00 ^b^	0.01 ± 0.00 ^b^	n.d.	n.d.	n.d.	n.d.	0.04 ± 0.00 ^b^	n.d.	0.07 ± 0.04 ^b^	n.d.
8	1-*O*-caffeoylquinic acid	n.d.	0.04 ± 0.00 ^d^	0.47 ± 0.02 ^a^	0.07 ± 0.00 ^c^	n.d.	0.05 ± 0.00 ^cd^	n.d.	0.06 ± 0.00 ^cd^	n.d.	0.06 ± 0.00 ^cd^	n.d.	0.12 ± 0.00 ^b^
9	caffeic acid	0.76 ± 0.01 ^a^	0.10 ± 0.00 ^e^	n.d.	0.04 ± 0.00 ^c^	0.53 ± 0.00 ^f^	0.03 ± 0.01 ^d^	0.55 ± 0.01 ^f^	0.03 ± 0.01 ^f^	0.55 ± 0.00 ^f^	0.04 ± 0.00 ^b^	0.50 ± 0.00 ^bc^	n.d.
10	4-*p*-coumaroylquinic acid	n.d.	0.05 ± 0.00 ^b^	n.d.	0.05 ± 0.00 ^b^	n.d.	0.03 ± 0.00 ^c^	n.d.	0.01 ± 0.00 ^d^	n.d.	0.02 ± 0.00 ^cd^	n.d.	0.07 ± 0.00 ^a^
11	procyanidin C1	0.09 ± 0.02 ^b^	n.d.	0.12 ± 0.01 ^ab^	n.d.	0.15 ± 0.00 ^a^	n.d.	0.13 ± 0.00 ^a^	n.d.	0.15 ± 0.00 ^a^	n.d.	n.d.	n.d.
12	5-feruloylquinic acid	2.43 ± 0.17 ^a^	0.52 ± 0.00 ^e^	1.41 ± 0.06 ^b^	0.36 ± 0.01 ^efg^	0.77 ± 0.00 ^d^	0.33 ± 0.02 ^fg^	0.51 ± 0.00 ^ef^	0.21 ± 0.00 ^gh^	0.24 ± 0.00 ^gh^	0.06 ± 0.00 ^h^	0.99 ± 0.01 ^c^	0.29 ± 0.01 ^g^
13	quercetin-3-*O*-neohesperidoside	0.28 ± 0.07 ^a^	0.05 ± 0.00 ^de^	0.15 ± 0.01 ^bc^	0.05 ± 0.00 ^de^	0.09 ± 0.00 ^cde^	0.03 ± 0.00 ^e^	0.07 ± 0.00 ^cde^	0.03 ± 0.00 ^e^	0.12 ± 0.00 ^bcd^	n.d.	0.18 ± 0.02 ^b^	0.04 ± 0.00 ^e^
14	quercetin-3-*O*-sambubioside	0.08 ± 0.01 ^cd^	n.d.	0.07 ± 0.00 ^d^	n.d.	0.11 ± 0.00 ^a^	n.d.	0.09 ± 0.00 ^bc^	n.d.	0.09 ± 0.00 ^ab^	n.d.	0.07 ± 0.00 ^cd^	n.d.
15	quercetin-3-*O*-galactoside	0.45 ± 0.05 ^b^	0.01 ± 0.00 ^e^	0.21 ± 0.01 ^d^	0.01 ± 0.00 ^e^	0.60 ± 0.00 ^a^	0.05 ± 0.00 ^e^	0.46 ± 0.01 ^b^	0.04 ± 0.00 ^e^	0.21 ± 0.00 ^d^	0.02 ± 0.00 ^e^	0.33 ± 0.00 ^c^	0.01 ± 0.00 ^e^
16	kaempferol-3-*O*-neohesperidoside	0.26 ± 0.01 ^b^	n.d.	0.07 ± 0.00 ^d^	n.d.	0.39 ± 0.00 ^a^	n.d.	0.25 ± 0.02 ^b^	n.d.	0.08 ± 0.00 ^d^	0.02 ± 0.00 ^e^	0.21 ± 0.00 ^c^	n.d.
17	3,6,7,2′,4′-pentamethylquercetagetin 3′-*O*-glucoside	0.04 ± 0.01 ^d^	0.03 ± 0.00 ^e^	0.10 ± 0.00 ^c^	0.01 ± 0.00 ^f^	0.17 ± 0.00 ^a^	0.02 ± 0.00 ^f^	0.14 ± 0.00 ^b^	0.02 ± 0.00 ^ef^	0.09 ± 0.00 ^c^	0.09 ± 0.00 ^c^	0.10 ± 0.00 ^c^	0.05 ± 0.00 ^d^
18	chrysosplenoside B	0.11 ± 0.02 ^a^	0.06 ± 0.00 ^b^	n.d.	0.01 ± 0.00 ^d^	n.d.	0.03 ± 0.00 ^cd^	n.d.	0.02 ± 0.00 ^d^	n.d.	0.02 ± 0.01 ^cd^	n.d.	0.05 ± 0.00 ^bc^
19	kaempferol-3-*O*-glucoside	0.31 ± 0.03 ^c^	n.d.	0.63 ± 0.02 ^a^	n.d.	0.62 ± 0.00 ^a^	n.d.	0.42 ± 0.01 ^b^	n.d.	0.13 ± 0.00 ^e^	n.d.	0.23 ± 0.00 ^d^	n.d.
20	quercetin-3-*O*-rhamnoside	0.35 ± 0.03 ^c^	n.d.	0.21 ± 0.01 ^d^	n.d.	0.60 ± 0.00 ^a^	0.02 ± 0.00 ^e^	0.44 ± 0.01 ^b^	0.01 ± 0.00 ^e^	0.18 ± 0.00 ^d^	0.01 ± 0.00 ^e^	0.40 ± 0.01 ^b^	n.d.
21	kaempferol-3-*O*-rhamnoside	0.37 ± 0.02 ^b^	n.d.	0.14 ± 0.01 ^c^	n.d.	0.51 ± 0.00 ^a^	n.d.	0.39 ± 0.00 ^b^	n.d.	0.15 ± 0.00 ^c^	n.d.	0.17 ± 0.00 ^c^	n.d.
22	kaempferol-3-*O*-(6″-acetyl)glucoside	0.05 ± 0.00 ^a^	n.d.	0.04 ± 0.00 ^a^	n.d.	0.04 ± 0.01 ^a^	0.02 ± 0.00 ^b^	n.d.	0.01 ± 0.00 ^b^	n.d.	n.d.^b^	n.d.	n.d.
23	oleanolic acid	0.14 ± 0.01 ^b^	0.06 ± 0.01 ^c^	0.22 ± 0.01 ^a^	0.08 ± 0.01 ^c^	0.16 ± 0.00 ^b^	0.09 ± 0.01 ^c^	0.09 ± 0.00 ^c^	0.08 ± 0.00 ^c^	0.08 ± 0.00 ^c^	0.07 ± 0.01 ^c^	0.08 ± 0.00 ^c^	0.07 ± 0.01 ^c^
24	ursolic acid	0.14 ± 0.00 ^c^	0.01 ± 0.00 ^d^	0.43 ± 0.02 ^a^	0.02 ± 0.00 ^d^	0.24 ± 0.00 ^b^	0.03 ± 0.00 ^d^	0.14 ± 0.00 ^c^	0.03 ± 0.00 ^d^	0.02 ± 0.00 ^d^	0.01 ± 0.00 ^d^	0.12 ± 0.00 ^c^	0.01 ± 0.00 ^d^

Note: n.d.—not detected. Results were the mean ± SD (n = 3) on a dried weight (g) of loquat basis. Values within each line followed by different letters indicate significant differences (*p* < 0.05) among different varieties and tissues according to Student *t* tests.

## Data Availability

All of the data is contained within the article and the Supplementary Materials.

## Supplementary Materials

The following supporting information can be downloaded at: https://www.mdpi.com/article/10.3390/antiox12101795/s1, Figure S1: Mouse body weight; Table S1: RT-PCR Primers.
