# Supplementary material for: Phenolics and Terpenoids Profiling in Diverse Loquat Fruit Varieties and Systematic Assessment of Their Mitigation of Alcohol-Induced Oxidative Stress
Source: Antioxidants (Basel). 2023 Sep 23;12(10):1795. doi: 10.3390/antiox12101795 (PMC10604257; doi:10.3390/antiox12101795)
Supplement: Supplementary file 1 [file antioxidants-12-01795-s001.zip › antioxidants-2624056-supplementary.pdf]

Table S1 RT-PCR Primers

Gene	Forward Primer (5' to 3')	Reverse Primer (3' to 5')
Nrf2	TCAGCAGCATCCTCTCCAC	GGTCACAGCCTTCAATAGTCC
Keap1	GGCGGCAGAAGAAGTCATC	TGGTGGTGGGAGTTCAAGG
Cul3	TCCCACCAGCACCAAGAC	CACCGCCAACACCAACC
β-Actin	CGACAGCAGTTGGTTGGAG	GGGAGGGTGAGGGACTTC

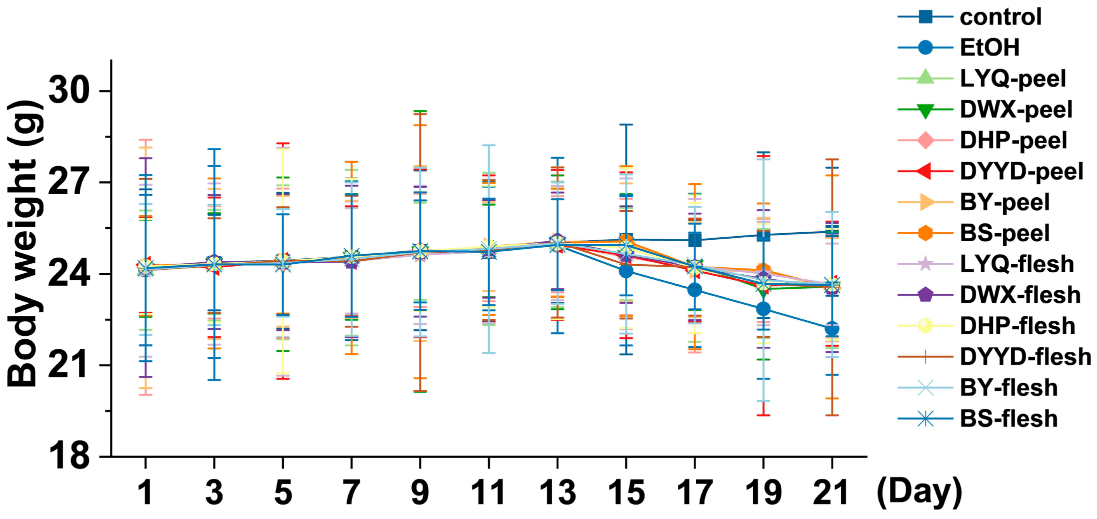

Figure S1 Mouse body weight
